# CD4 T cell–activating neoantigens enhance personalized cancer vaccine efficacy

**DOI:** 10.1172/jci.insight.174027

**Published:** 2023-12-08

**Authors:** Amanda L. Huff, Gabriella Longway, Jacob T. Mitchell, Lalitya Andaloori, Emily Davis-Marcisak, Fangluo Chen, Melissa R. Lyman, Rulin Wang, Jocelyn Mathew, Benjamin Barrett, Sabahat Rahman, James Leatherman, Mark Yarchoan, Nilofer S. Azad, Srinivasan Yegnasubramanian, Luciane T. Kagohara, Elana J. Fertig, Elizabeth M. Jaffee, Todd D. Armstrong, Neeha Zaidi

**Affiliations:** 1 Johns Hopkins Convergence Institute and; 2 Johns Hopkins Bloomberg Kimmel Institute for Immunotherapy, Johns Hopkins School of Medicine, Baltimore, Maryland, USA.; 3 Department of Oncology, Sidney Kimmel Comprehensive Cancer Center, Johns Hopkins University, Baltimore, Maryland, USA.; 4 Department of Human Genetics, Johns Hopkins School of Medicine, Baltimore, Maryland, USA.; 5 inHealth Precision Medicine Program,; 6 Department of Applied Mathematics and Statistics, and; 7 Department of Biomedical Engineering, Johns Hopkins School of Medicine, Baltimore, Maryland, USA.

**Keywords:** Immunology, Oncology, Cancer immunotherapy, MHC class 2, T cells

## Abstract

Personalized cancer vaccines aim to activate and expand cytotoxic antitumor CD8^+^ T cells to recognize and kill tumor cells. However, the role of CD4^+^ T cell activation in the clinical benefit of these vaccines is not well defined. We previously established a personalized neoantigen vaccine (PancVAX) for the pancreatic cancer cell line Panc02, which activates tumor-specific CD8^+^ T cells but required combinatorial checkpoint modulators to achieve therapeutic efficacy. To determine the effects of neoantigen-specific CD4^+^ T cell activation, we generated a vaccine (PancVAX2) targeting both major histocompatibility complex class I– (MHCI-) and MHCII-specific neoantigens. Tumor-bearing mice vaccinated with PancVAX2 had significantly improved control of tumor growth and long-term survival benefit without concurrent administration of checkpoint inhibitors. PancVAX2 significantly enhanced priming and recruitment of neoantigen-specific CD8^+^ T cells into the tumor with lower PD-1 expression after reactivation compared with the CD8^+^ vaccine alone. Vaccine-induced neoantigen-specific Th1 CD4^+^ T cells in the tumor were associated with decreased Tregs. Consistent with this, PancVAX2 was associated with more proimmune myeloid-derived suppressor cells and M1-like macrophages in the tumor, demonstrating a less immunosuppressive tumor microenvironment. This study demonstrates the biological importance of prioritizing and including CD4^+^ T cell–specific neoantigens for personalized cancer vaccine modalities.

## Introduction

Neoantigens arise from nonsynonymous somatic mutations, alternative RNA splicing, or dysregulated posttranslational modification during tumorigenesis and serve as unique tumor-specific immunologic targets for personalized cancer vaccines (1, 2). Multiple preclinical and clinical studies have shown that neoantigen-targeted vaccines can activate neoantigen-specific T cells that recognize and kill tumor cells expressing the neoantigens (3–10). To generate a personalized neoantigen-targeted vaccine, tumor tissue is obtained from a resected tumor, which is then subject to whole-exome and RNA-Seq to identify unique patient-tumor–specific mutations. In silico algorithms, such as NetMHC, are then utilized to predict which neoantigens have a strong binding affinity to a patient’s human leukocyte antigen (HLA) class I alleles (11). The generated neoantigen-targeted vaccines are often delivered using 20–30 mer synthetic long peptides (SLPs), along with an immune adjuvant, such as Poly-ICLC, a synthetic dsRNA that stimulates a robust type I IFN response through TLR3 (12, 13). The use of SLPs allows for processing and presentation of both HLA class I (8–11 mer) and HLA class II (12–15 mer) epitopes to stimulate CD8^+^ and CD4^+^ T cells, respectively. While most studies to date have focused on eliciting neoantigen-specific CD8^+^ T cells that trigger a direct cytotoxic antitumor effect, CD4^+^ T cell responses have also been observed to these SLP vaccines (3–6, 10). Despite a broad role for CD4^+^ T cells in antitumor immunity, the relative importance of eliciting neoantigen-specific CD4^+^ T cells in the context of a therapeutic vaccine have not been elucidated.

Here, we explored the role of neoantigen-specific CD4^+^ T cell responses in the context of a personalized neoantigen vaccine. We previously designed and validated a personalized cancer neoantigen vaccine (PancVAX), against the murine pancreatic cancer cell line Panc02 (14). PancVAX was designed using a standard, clinically utilized pipeline to predominantly activate neoantigen-specific CD8^+^ T cells. We showed that, while PancVAX alone did lead to transient tumor regression, combination therapy with immune checkpoint inhibitor (ICI) and costimulatory agonists was required for a durable antitumor response. In subsequent studies, we found that PancVAX either alone or in combination with ICIs did not lead to significant activation of CD4^+^ T cell responses, thereby providing a unique opportunity to better understand how inclusion of CD4^+^ T cell–specific neoantigens influences the efficacy of a personalized vaccine platform. To test the effects of concomitant CD4^+^ T cell activation by our neoantigen vaccine, we enriched PancVAX with SLPs that were predicted to bind murine major histocompatibility complex (MHC) class II and evoke a CD4^+^ T cell response. We hypothesized that enriching a personalized vaccine with CD4^+^ T cell–specific neoantigens would improve the quality of the CD8^+^ T cell response and result in enhanced antitumor immunity.

## Results

### The PancVAX personalized neoantigen vaccine platform lacks activation of CD4^+^ T cells.

**Figure 1:**
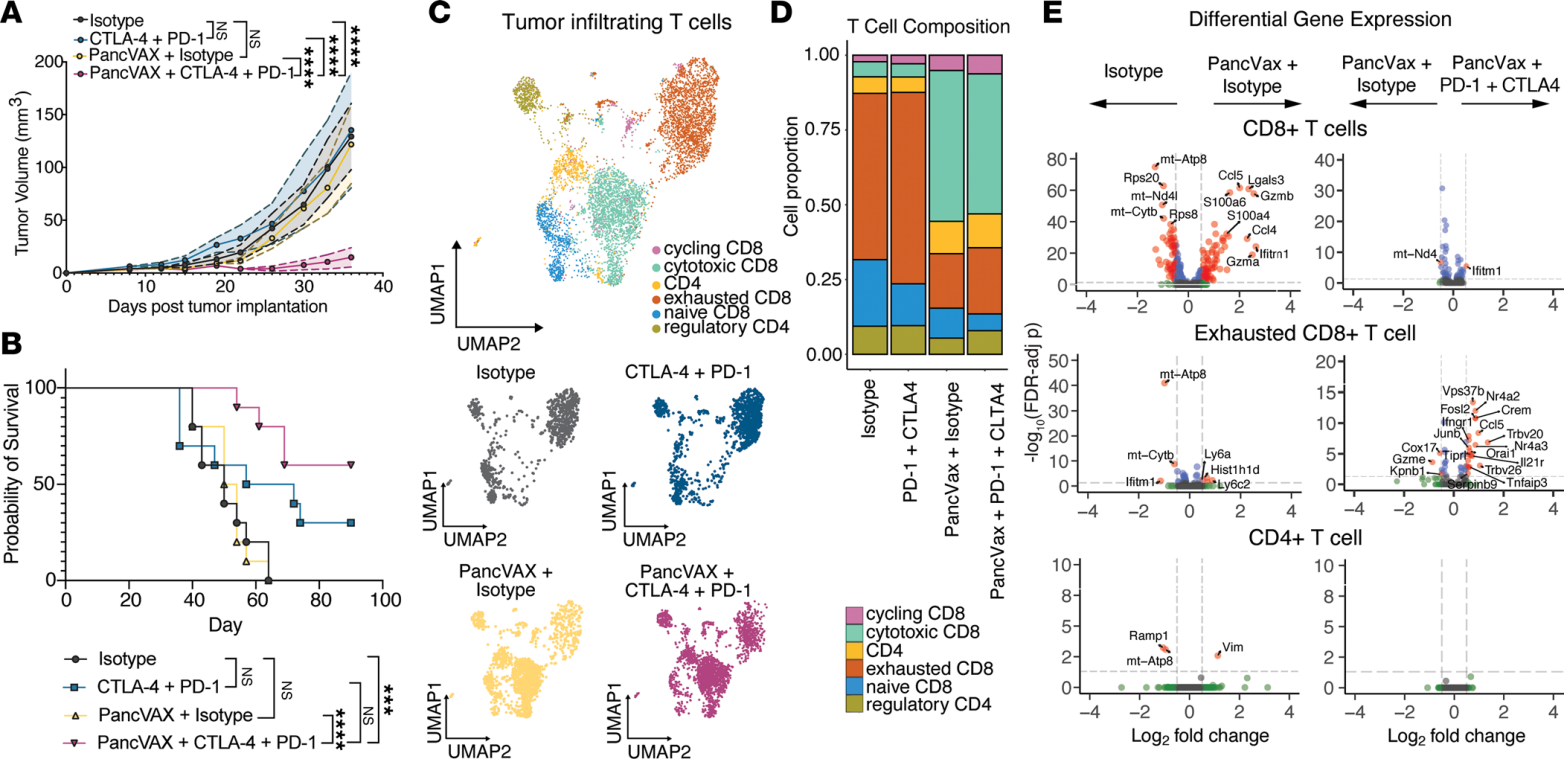
PancVAX plus ICIs elicit antitumoral CD8^+^ T cell immunity with minimal effect on CD4^+^ T cells. C57BL/6 mice were implanted with 1 × 10^6^ Panc02s s.c. and vaccinated with PancVAX, s.c. tail base on days 12 and 19. At day 12, mice received 100 µg anti–PD-1, i.p., or isotype twice weekly with 2 doses of 100 µg anti–CTLA-4 or isotype, i.p., on days 15 and 19. (**A**) Tumor volumes were measured in mice treated with isotype (gray), anti–CTLA-4, and–PD-1 (blue), PancVAX with isotype (yellow), and PancVAX with anti–CTLA-4 and PD-1 (magenta). Shaded regions represent data as mean ± SEM. *P* values derived from 2-way ANOVA with Tukey’s multiple-comparison test (****P ≤ 0.0001). (**B**) Survival analysis. *P* values derived from Log-rank (Mantel-Cox) (***P ≤ 0.001, ****P ≤ 0.0001). (**C**) Uniform manifold approximation plot (UMAP) of 6,423 T cells from Panc02 tumors (day 35). Cells annotated as cycling T cells (pink), cytotoxic CD8^+^ T cells (teal), effector CD4^+^ T cells (gold), exhausted CD8^+^ T cells (orange), naive CD8^+^ T cells (blue), and regulatory T cells (olive). UMAP of distribution of T cell populations across groups: Isotype control (gray), anti–CTLA-4, and anti–PD-1 (blue), PancVAX with isotype (yellow), and PancVAX with anti–CTLA-4 and PD-1 (magenta). (**D**) Stacked bar plot of the proportions of T cell phenotype across groups. (**E**) Volcano plots of MAST tests for differential expression between Isotype control- and PancVAX-treated, PancVAX + Isotype–treated, and PancVAX + anti–PD-1 + anti–CTLA-4–treated CD8^+^ T cells in total (top), exhausted CD8^+^ T cells (middle), and CD4^+^ T cells (bottom). Genes with significant FDR-adjusted *P* values (adjusted *P* < 0.05) and average log_2_-fold changes (|log_2_FC| > 0.5) are colored red.

We previously generated PancVAX by screening immunogenicity of SLPs for 245 neoantigens identified in the Panc02 line that were predicted to bind 2 MHCI alleles expressed by Panc02 cells, H2-K^b^ or H2-D^b^, with a binding affinity of < 1,000 nM (14) (Supplemental Table 1; supplemental material available online with this article; https://doi.org/10.1172/jci.insight.174027DS1). Consistent with our previous work, mice treated with PancVAX delivered in combination with a STING agonist ADU-S100 and dual ICIs, anti–PD-1 and anti–CTLA-4 antibodies, displayed a significant improvement in the control of early tumor growth compared with vaccine alone or ICIs alone (Figure 1A). Additionally, only PancVAX with ICIs resulted in a significant improvement in long-term survival compared with isotype treatment only (Figure 1, A and B). Single-cell RNA-Seq of tumor-infiltrating lymphocytes (TILs) isolated from tumors after PancVAX or PancVAX with combination ICI treatment identified notable differences in cytotoxic T cell infiltration compared with PancVAX plus isotype control (Supplemental Figure 1 and Figure 1, C–E). Differential gene expression analysis indicated significant increases in inflammatory and effector gene expression, such as granzyme B (*Gzmb*) and CC-chemokine ligand 5 (*Ccl5*), in CD8^+^ T cells within the tumor microenvironment (TME) after PancVAX compared with isotype control–treated tumors (Figure 1E, upper). As expected, combination with ICI therapy induced significant upregulation of a proinflammatory gene expression profile within the exhausted T cell population, such as IFN-γ receptor (*Ifnar1*), IL-21 receptor (*Il21r*), and CCL5 (*Ccl5*), likely contributing to improved therapeutic efficacy (Figure 1E, middle). Notably, there was no CD4^+^ T cell activation in PancVAX-treated mice compared with isotype control–treated mice or in the context of PancVAX with ICI, despite vaccination with 20 mer SLPs that could allow for CD4^+^ T cell priming (Figure 1E, lower). We therefore reasoned that this model provides a unique opportunity to learn how incorporation of CD4^+^ T cell antigens into a personalized neoantigen vaccine may contribute to antitumor immunity and possibly improve therapeutic efficacy.

### Identification of immunogenic MHCII neoantigens expressed by Panc02 cells.

**Figure 2:**
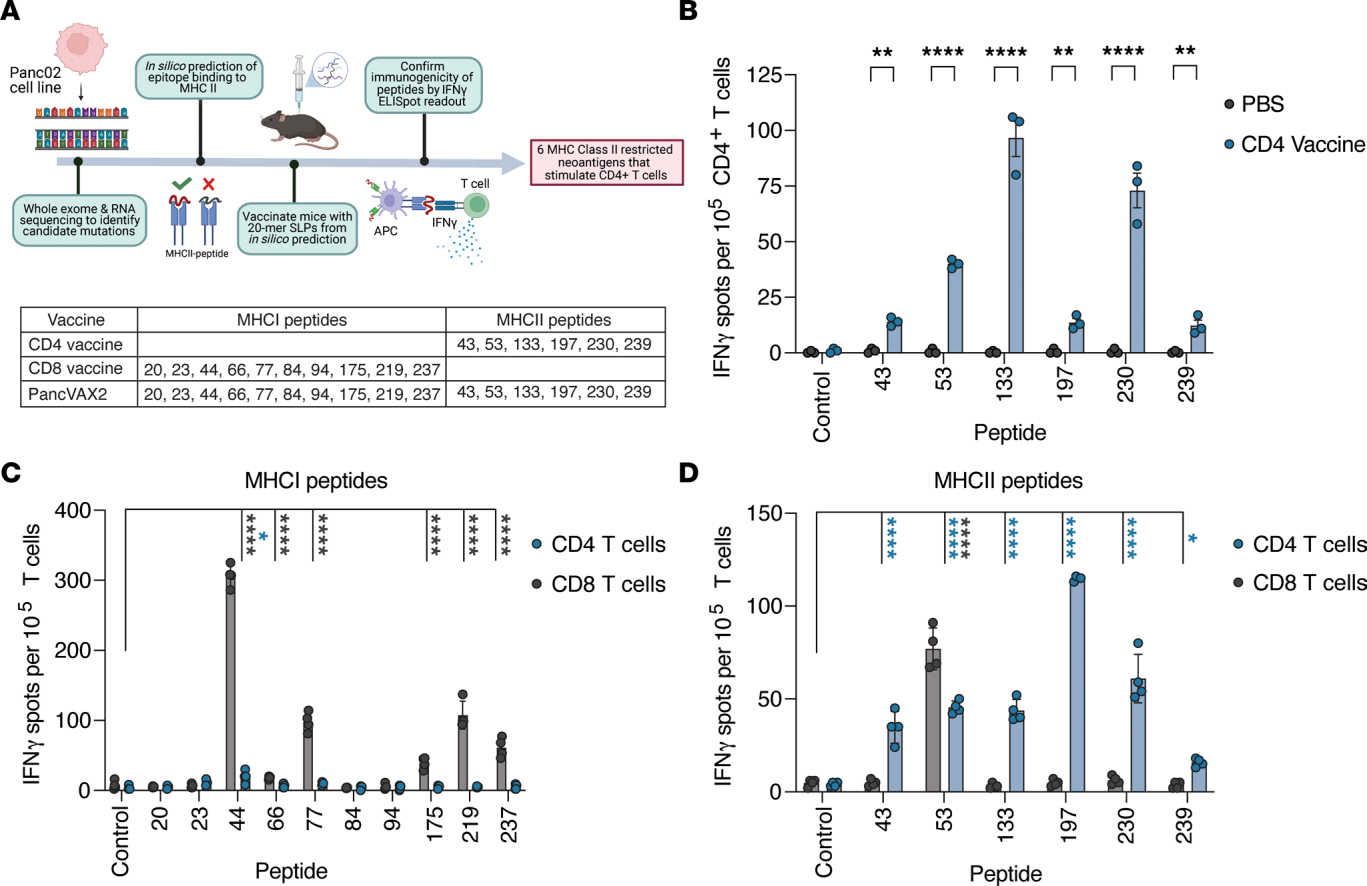
Murine pancreatic cancer MHCII predicted neoantigens are immunogenic in non–tumor-bearing mice. (**A**) Pipeline to identify predicted MHCII restricted neoantigens that stimulate a CD4^+^ T cell response in vivo. (**B**) Non–tumor bearing C57BL/6 mice were vaccinated with 20 mer SLPs predicted to bind to H2-IA^b^ on days 0 and 7 (*n* = 3). Splenocytes from vaccinated mice were pooled on day 14, and CD4^+^ T cells were isolated using magnetic bead negative isolation kits. Isolated CD4^+^ T cells were cocultured overnight at a 1:1 ratio with I-A^b^–expressing T-2 APCs pulsed with MHCII-specific peptides in an IFN-γ capture plate and assessed for reactivity by ELISpot the following day. The 20 mer neoepitopes that produced a significant immune response in the CD4 vaccinated group over the untreated group are displayed. Symbols represent technical replicates. To confirm CD4 and CD8 T cell specificity to each peptide, non–tumor-bearing C57BL/6 mice were vaccinated with PancVAX2 on days 0 and 7 (*n* = 4). (**C** and **D**) Splenocytes from vaccinated mice were isolated and pooled on day 14, and CD4^+^ and CD8^+^ T cells were separately sorted using magnetic bead negative isolation kits and cocultured overnight at a 1:1 ratio with matured murine BMDCs that had been pulsed with 5 μg/mL MHCI-specific (**C**) or MHCII-specific (**D**) peptides in an IFN-γ capture plate and assessed for reactivity by ELISpot the following day. Data are shown as mean ± SD. Significance was calculated by 2-way ANOVA followed by Sidak’s multiple-comparison test. *P ≤ 0.05, **P ≤ 0.01,****P ≤ 0.0001. Significance values for CD8 T cell cultures (black asterisks) and CD4 T cell cultures (blue asterisks) are shown.

We previously identified 878 nonsynonymous mutations in the murine pancreatic cancer Panc02 cell line and determined that 269 of these were predicted to bind to MHCI (14). Of these, 245 neoantigens were initially manufactured as SLPs and screened for CD8^+^ T cell immunogenicity in vivo. To include CD4^+^ T cell–specific neoantigens in the Panc02 vaccine, we used a publicly available database, Immune Epitope Database (IEDB), to rank the binding affinity of these neoantigens to murine I-A^b^, the MHCII allele expressed by Panc02 (15). The neoepitopes that displayed a binding affinity greater than the 50th percentile for I-A^b^ were tested for in vivo immunogenicity for CD4^+^ T cell responses (Figure 2A). WT C57BL/6 mice were vaccinated s.c. with 20 mer SLPs on days 0 and 7. At day 14, IFN-γ–producing CD4^+^ T cells in response to neoantigen peptide restimulation were quantitated by ELISpot, with OVAII (SLKISQAVHAAHAEINEAGR) as a negative control. We identified 6 immunogenic MHCII neoantigens in the Panc02 model that elicited CD4^+^ T cell responses (peptides 43, 53, 133, 197, 230, 239; Supplemental Table 2 and Figure 2, A and B). As observed previously for MHCI predicted neoantigens, the predicted binding affinity of MHCII neoantigens did not always correlate with the magnitude of response to each neoantigen (Supplemental Table 2 and Figure 2B) (16). For example, peptides 53 and 230 were predicted to have a low binding affinity (>1,000 nM) but were highly immunogenic in vivo.

We combined the 6 CD4^+^ T cell–specific neoantigens (CD4 vaccine) with our previously identified CD8^+^ T cell neoantigens (CD8 vaccine) to generate a second-generation vaccine, PancVAX2. Given that SLPs would theoretically elicit both CD4^+^ and CD8^+^ T cell responses, we next confirmed CD4^+^ or CD8^+^ T cell reactivity to each neoantigen. CD4^+^ or CD8^+^ T cells isolated from splenocytes of non–tumor-bearing mice vaccinated with PancVAX2 were cocultured with peptide pulsed bone marrow–derived DCs (BMDCs) and assessed for IFN-γ secretion by ELISpot (Supplemental Figure 2). Consistent with our previous data, restimulation with the MHCI epitopes elicited a predominant IFN-γ response from isolated CD8^+^ T cells for all epitopes with a minimal CD4^+^ T cell response observed against peptide 44 (Figure 2C). These data were consistent with a lack of strong CD4^+^ T cell activation from our PancVAX platform observed in our single-cell RNA-Seq data set (Figure 1E). In addition, the MHCII epitopes predominantly triggered an IFN-γ response from isolated CD4^+^ T cells, while peptide 53 activated both CD4^+^ and CD8^+^ T cells (Figure 2D). We next examined whether inclusion of the CD4^+^ T cell–activating neoantigens enhanced the immunogenicity of vaccine-induced, neoantigen-specific CD8^+^ T cells. We observed that mice vaccinated with PancVAX2 primed significantly greater responses to several CD8^+^ vaccine epitopes compared with mice vaccinated with the CD8^+^ T cell epitopes only, consistent with the role of CD4^+^ T cell help in CD8^+^ T cell priming (Supplemental Figure 3).

### PancVAX2 improves control of tumor growth and survival.

**Figure 3:**
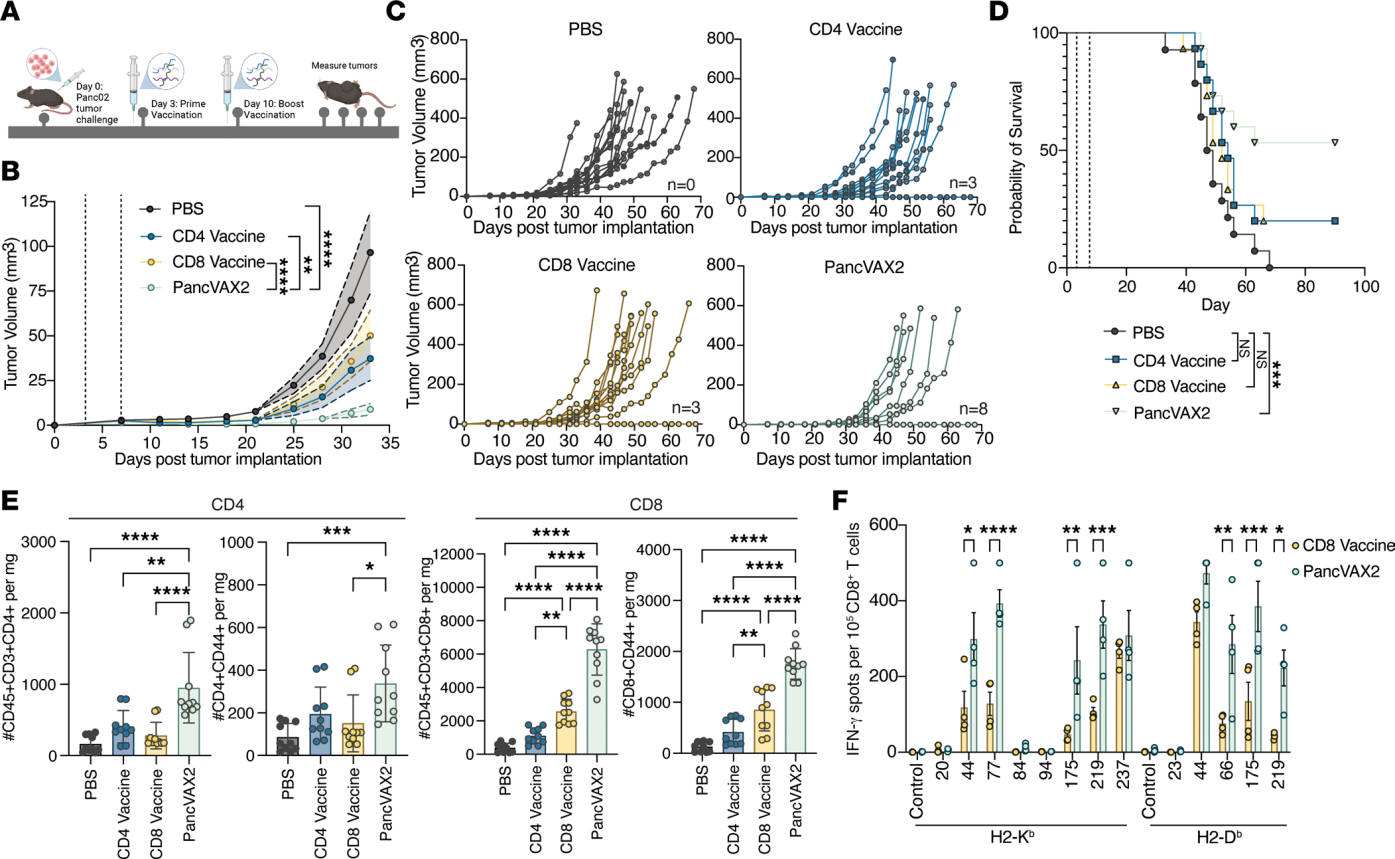
PancVAX2 improves control of tumor growth and long-term survival associated with an increase in tumor infiltrating T cells. (**A**) C57BL/6 mice were implanted with 3 × 10^6^ Panc02s, s.c., on day 0. Mice were vaccinated s.c. at base of tail on days 3 and 10 with PBS (*n* = 14) or CD4 Vaccine, CD8 Vaccine, or PancVAX2 (n = 15). (**B**) Average tumor volumes were plotted until day 33. Data shown as mean ± SEM. Significance on day 33 calculated by 2-way ANOVA followed by Sidak’s multiple-comparison test. (**C**) Spider plots of individual growth curves. Number of tumor free mice at day 70: PBS (*n* = 0), CD4 vaccine (*n* = 3), CD8 vaccine (*n* = 3), PancVAX2 (*n* = 8). (**D**) Kaplan-Meier survival curves. Vaccination time points shown as gray dashed line. Significance calculated by Log-rank (Mantel-Cox) test. (**E**) C57BL/6 mice implanted with 3 × 10^6^ Panc02s, s.c., on day 0. Mice were vaccinated s.c. on day 14 and 21 with PBS, CD4 Vaccine, CD8 Vaccine, or PancVAX2 (*n* = 5). On day 28, tumors were harvested, dissociated into a single-cell suspension, and analyzed by flow cytometry. Total CD4 and CD8 or activated (CD44^+^) CD4 and CD8 populations per mg tumor were quantified from the CD45^+^CD3^+^CD19^-^NK1.1^–^ population. Symbols represent technical duplicate of individual mice. (**F**) C57BL/6 mice were implanted with 3 × 10^6^ Panc02s, s.c., on day 0. Mice were vaccinated s.c. on day 28 with PBS or CD4 Vaccine, CD8 Vaccine, or PancVAX2 (*n* = 4). On day 35, tumors were harvested, and CD8 T cells were isolated and cocultured in an IFN-γ capture plate at a 1:1 ratio with T2-H2-Kb or T2-H2-Db APCs pulsed with 5 µg/mL MHCI peptides for 24 hours. Symbols represent individual mice. Data are shown as mean ± SD. Significance was calculated by 2-way ANOVA followed by Sidak’s multiple-comparison test. *P ≤ 0.05, **P ≤ 0.01, ***P ≤ 0.001, ****P ≤ 0.0001.

To test the therapeutic efficacy of PancVAX2, mice bearing s.c. Panc02 tumors were vaccinated with PancVAX2, CD8 vaccine, CD4 vaccine, or PBS (untreated) on days 3 and 10 (Figure 3A). Average tumor volume growth after treatment indicated mice vaccinated with PancVAX2 experienced a significant decrease in average tumor volume compared with mice treated with PBS, CD4 vaccine, or CD8 vaccine (Figure 3, B and C). PancVAX2 treatment led to the greatest number of tumor-free mice at 90 days after vaccination (8 of 15) compared with those treated with CD4 vaccine (3 of 15), CD8 vaccine (3 of 15), or PBS (0 of 15) (Figure 3C). This improved response with PancVAX2 vaccination on tumor growth translated into a survival benefit that was significant and sustained for at least 3 months (Figure 3D). No survival benefit was observed in mice treated with the CD8 vaccine only, consistent with our previous data demonstrating the need for codelivery of a costimulatory agonist and ICI (14).

To assess the relative composition of CD8^+^ and CD4^+^ T cells in the tumors, we performed flow cytometry on TILs isolated from all 4 groups of vaccinated mice. We found a greater increase in the number of infiltrating CD4^+^ and CD8^+^ T cells in the PancVAX2 group compared with mice vaccinated with either CD4 or CD8 vaccines or PBS (Figure 3E and Supplemental Figure 4). Further analysis of the CD44^+^ (activated) subsets of both CD4^+^ and CD8^+^ T cells showed similar results. In addition, and expectedly, there were significant increases in CD8^+^ T cells with the CD8 vaccine, with a trend toward increases in CD4^+^ T cells with the CD4^+^ vaccine when compared with PBS.

To determine if the observed increased CD8^+^ T cell infiltrates were specific to the vaccine neoantigens, intratumoral CD8^+^ T cells from mice treated with the CD8 vaccine or PancVAX2 were isolated and restimulated with the 8 individual MHCI SLPs in the context H2-K^b^ or H2-D^b^. Notably, we found PancVAX2-treated mice had a significant increase in neoantigen-specific CD8^+^ T cells within the tumor against neoantigens 44, 66, 77, 175, and 219 as measured by IFN-γ secretion (Figure 3F). This suggests that concomitant activation of CD4^+^ T cells by a neoantigen vaccine platform not only improves priming of CD8 epitope responses but also plays a vital role in optimizing the trafficking of neoantigen-specific CD8^+^ T cells into the tumor.

### PancVAX2 induces a Th1 CD4^+^ T cell response and enhances infiltration of less exhausted cytotoxic CD8^+^ T cells.

**Figure 4:**
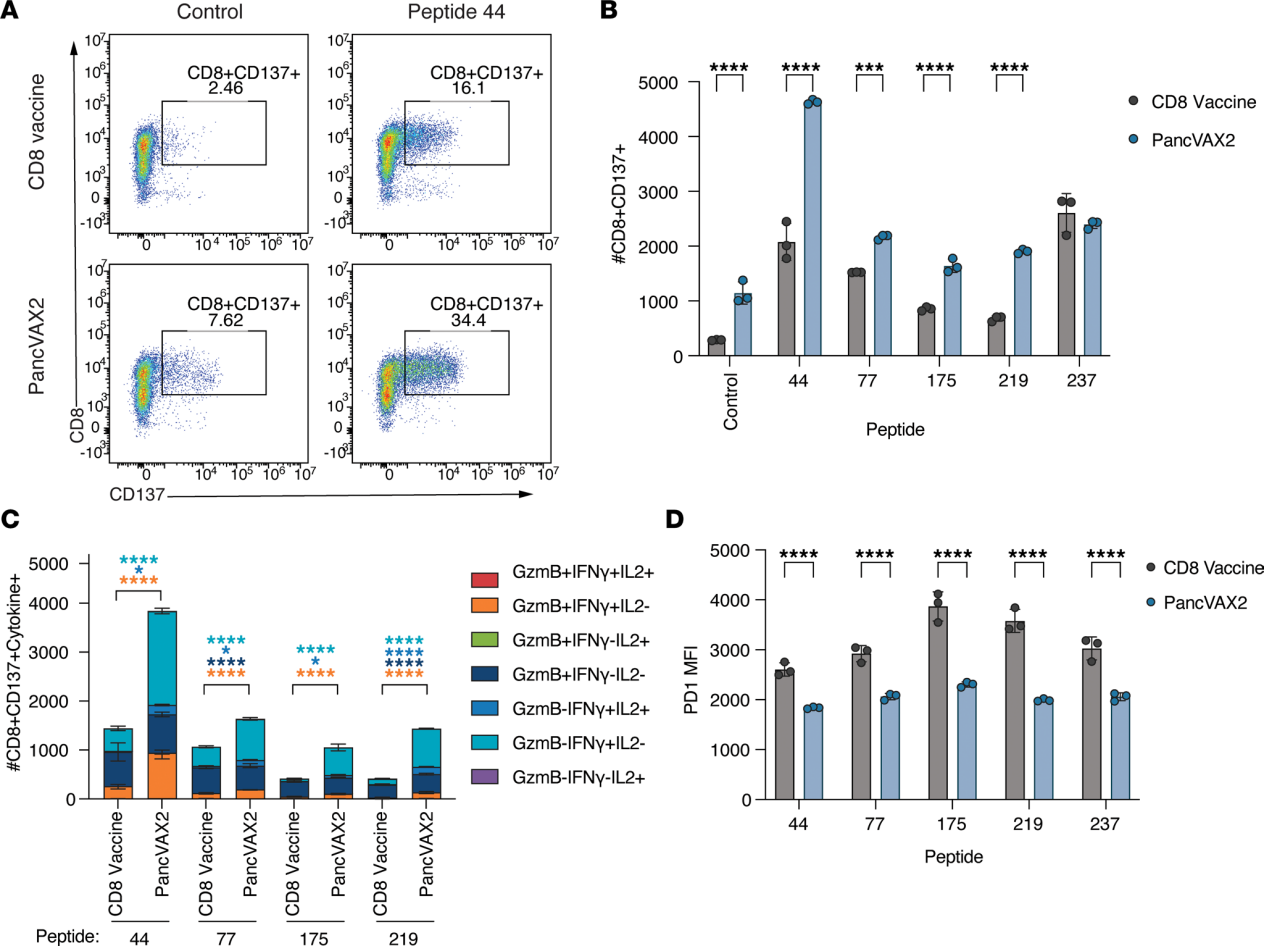
PancVAX2 enhances infiltration of neoantigen-specific cytotoxic CD8^+^ T cells into the tumor with a less exhausted phenotype. C57BL/6 mice were implanted on the right flank, s.c., with 3 × 10^6^ Panc02 cells on day 0. Mice were vaccinated s.c. at the base of the tail on day 14 and 21 with CD8 vaccine or PancVAX2 (*n* = 5). On day 28, tumors were harvested and pooled within treatment groups and dissociated into a single-cell suspension, and CD8^+^ T cells were isolated by magnetic positive selection kit. Isolated CD8^+^ T cells were cocultured overnight at a 1:1 ratio with T2-H2-K^b^ APCs pulsed with 5 μg/mL MHCI-specific peptides. Cocultures were then stained for activation marker and effector cytokine expression by flow cytometry analysis. (**A**) Representative gating of CD8 and CD137 marker expression on tumor infiltrating T cells cocultured with T-2 APCs pulsed with OVA peptide (control) or peptide 44. (**B**) Quantification of CD137 upregulation on tumor infiltrating CD8^+^ T cells cocultured with immunogenic CD8 epitopes. (**C**) Cytokine expression (GzmB, IFN-γ, and IL-2) from activated (CD137^+^) CD8^+^ T cells that stimulated a greater response in PancVAX2-treated tumors than in CD8 vaccinated tumors. (**D**) PD-1 expression measured by median fluorescence intensity (MFI) on activated (CD137^+^) CD8^+^ T cells after peptide restimulation. Symbols represent technical triplicates of pooled CD8^+^ T cells. Data are shown as mean ± SD. Significance was calculated by 2-way ANOVA followed by Sidak’s multiple-comparison test. *P ≤ 0.05, ***P ≤ 0.001, ****P ≤ 0.0001.

We next immunophenotyped the PancVAX2-induced, neoantigen-specific CD8^+^ T cells derived from Panc02 tumors. CD8^+^ T cells were isolated from tumors of mice vaccinated with the CD8 vaccine or PancVAX2 and cocultured overnight with antigen presenting cells (APCs) pulsed with the H2-K^b^ dominant epitopes and analyzed by flow cytometry for CD137, also known as 4-1BB, a potent costimulatory molecule upregulated after T cell recognition of cognate peptide-MHC (Figure 4A). Consistent with Figure 3E, mice vaccinated with PancVAX2 displayed a significant increase in CD137^+^CD8^+^ T cells within the tumor after vaccination compared with mice receiving the CD8 vaccine (Figure 4B). We also observed a significant upregulation of CD137^+^CD8^+^ T cell populations in response to restimulation with all 5 SLPs in both vaccine groups (Figure 4B). However, for 4 of the 5 SLPs tested (peptides 44, 77, 175, and 219), the response magnitude with PancVAX2 was significantly greater than that with the CD8 vaccine (Figure 4B). Within this neoantigen-responsive population, there were significantly more polyfunctional CD8^+^ T cells expressing cytotoxic effector signatures GzmB^+^IFN-γ^+^, IFN-γ^+^IL-2^+^, GzmB^+^, or IFN-γ^+^ in mice vaccinated with PancVAX2 (Figure 4C). We also noted that, in response to peptide restimulation, the activated CD137^+^CD8^+^ T cells in the PancVAX2 group expressed significantly lower levels of PD-1, despite greater CD137 expression compared with activated T cells from the CD8 vaccine, suggesting a less exhausted phenotype in the PancVAX2-induced, neoantigen-specific CD8^+^ T cell population (Figure 4D).

**Figure 5:**
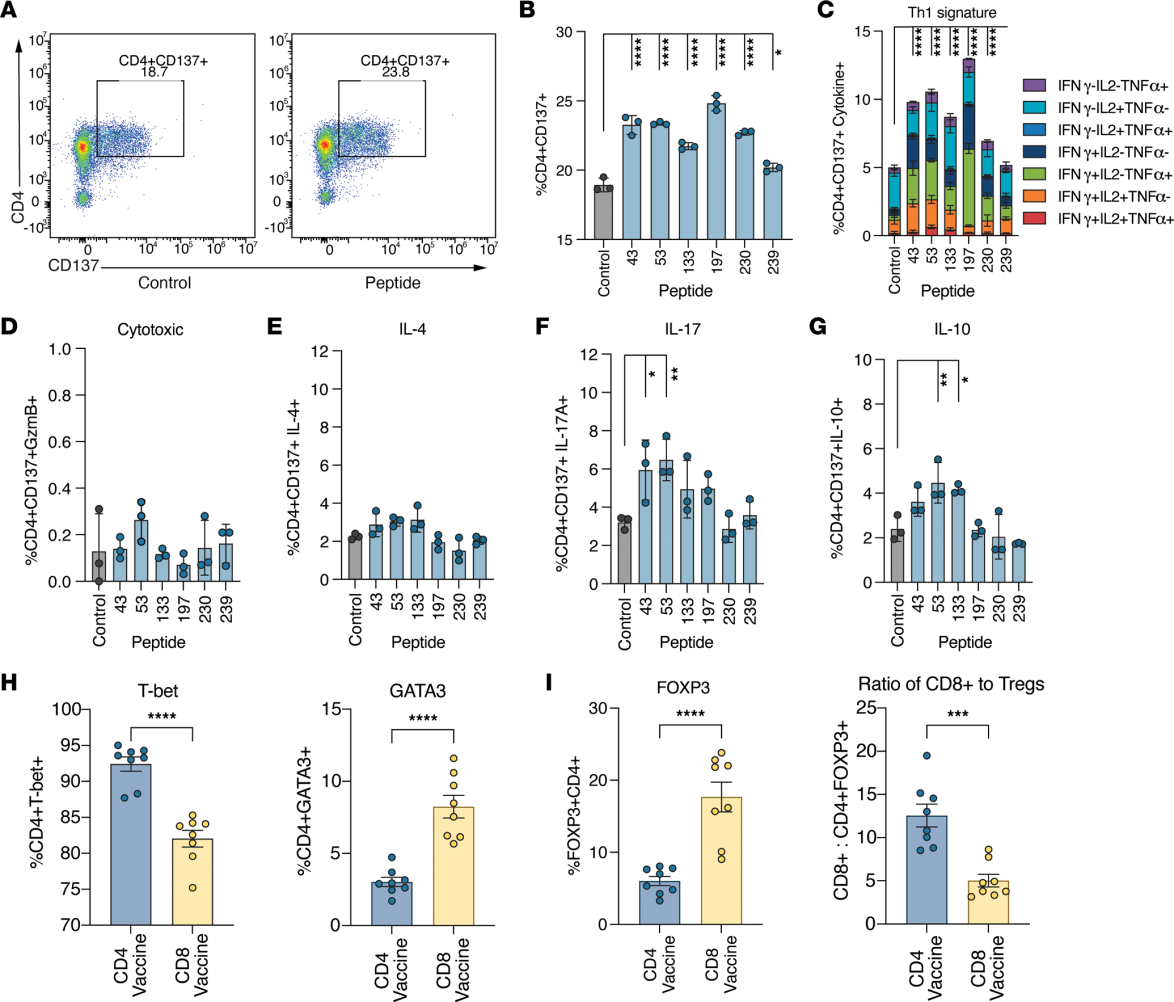
PancVAX2 recruits neoantigen-specific Th1 CD4^+^ T cells into the tumor and reduces infiltration of Tregs into the tumor. C57BL/6 mice were implanted with 3 × 10^6^ Panc02 cells, s.c., on day 0. Mice were vaccinated s.c. at the base of the tail on day 14 and 21 with PancVAX2 (*n* = 5). On day 28, tumors were harvested and pooled and dissociated into a single-cell suspension, and isolated CD4 T cells were cocultured overnight at a 1:1 ratio with T2-H2-IA^b^ APCs pulsed with 5 µg/mL MHCII peptides. (**A**) Representative gating of CD4 and CD137 expression from intratumoral CD3^+^ cells after cocultured with OVAII peptide (control) or peptide 43. (**B**) Quantification of CD4^+^CD137^+^ T cells following restimulation with each MHCII-specific peptide. (**C**–**G**) Activated (CD137^+^) CD4 T cells were then assessed for Th1-like cytokine expression (IFN-γ, IL-2, and TNF-α) (**C**), cytotoxic effector cytokine expression (GzB) (**D**), Th2-like cytokine expression (IL-4) (**E**), Th17-like cytokine expression (IL-17) (**F**), and Treg activity (IL-10) (**G**). Symbols represent technical triplicates from pooled CD4 T cells. Data are shown as mean ± SD. Significance was calculated by 2-way ANOVA followed by Sidak’s multiple-comparison test. (**H**) C57BL/6 mice were implanted on the right flank, s.c, with 3 × 10^6^ Panc02 cells on day 0. Mice were vaccinated s.c. at the base of the tail on day 14 and 21 with CD4 Vaccine or CD8 Vaccine (*n* = 4). On day 28, tumors were harvested, dissociated into a single-cell suspension, and stained for flow cytometry analysis. CD45^+^CD3^+^CD4^+^ populations were assessed for the proportion of cells expressing transcription factors T-bet (Th1, left) or GATA3 (Th2, right). (**I**) Proportion of FOXP3^+^CD4^+^ T cells (left) and the ratio of CD45^+^CD3^+^CD8^+^ T cells to FOXP3^+^CD4^+^ T cells (right). Symbols represent technical duplicate of individual mice. Data are shown as mean ± SD. Significance was calculated by a 2-tailed unpaired *t* test. *P ≤ 0.05, **P ≤ 0.01, ***P ≤ 0.001, ****P ≤ 0.0001.

To confirm the presence and determine the phenotype of the vaccine-induced CD4^+^ T cells within the TME that contributed to improved neoantigen-specific CD8^+^ T cell infiltration, CD4^+^ T cells were isolated from the tumors of PancVAX2 vaccinated mice and cocultured overnight with MHCII peptide-pulsed APCs. After peptide restimulation, a significant increase in activated CD137^+^CD4^+^ T cells was observed for every CD4 vaccine neoantigen (Figure 5, A and B) — confirming that neoantigen-specific CD4^+^ T cells infiltrated Panc02 tumors following PancVAX2. There was an increase in CD4^+^ T cells producing IFN-γ, IL-2, and TNF-α, indicating a Th1-like CD4^+^ T cell function (Figure 5C). Furthermore, we found that the neoantigen-specific CD4^+^ T cells did not express GzmB, suggesting that these CD4^+^ T cells did not exert direct antitumor cytotoxicity, although MHCII upregulation on Panc02 cells was observed after IFN-γ treatment in vitro (Figure 5D and Supplemental Figure 5). IL-4 secretion was not detected after peptide restimulation, confirming that a Th2 CD4^+^ T cell response was not generated by the vaccine (Figure 5E). Instead, there was an increase in IL-17 in response to peptides 43 and 53, suggesting a polyfunctional response to these epitopes associated with high inflammatory function (Figure 5F). IL-10 secretion was also observed in response to peptides 53 and 133, suggesting an initial immunosuppressive role for these antigen-reactive CD4^+^ T cells (Figure 5G). However, these IL-10^+^CD4^+^ T cells were also IFN-γ^+^ (Supplemental Figure 6), more consistent with a canonical Th1 cytokine signature (17). Consistent with this Th1 antigen–specific cytokine profile, mice vaccinated with the CD4 vaccine only had a significantly higher proportion of Tbet^+^CD4^+^ Th1 cells and significantly lower GATA3^+^CD4^+^ Th2 cell population within the tumor compared with mice vaccinated with the CD8 vaccine (Figure 5H). Additionally, the CD4 vaccine was associated with a lower frequency of FOXP3^+^ Tregs and, thus, a higher ratio of CD8^+^ T cells/Tregs within the TME, indicating an important role in CD4^+^ T cell responses in remodeling the immunosuppressive TME (Figure 5I).

### PancVAX2 reprograms a proimmune myeloid compartment within the TME.

**Figure 6:**
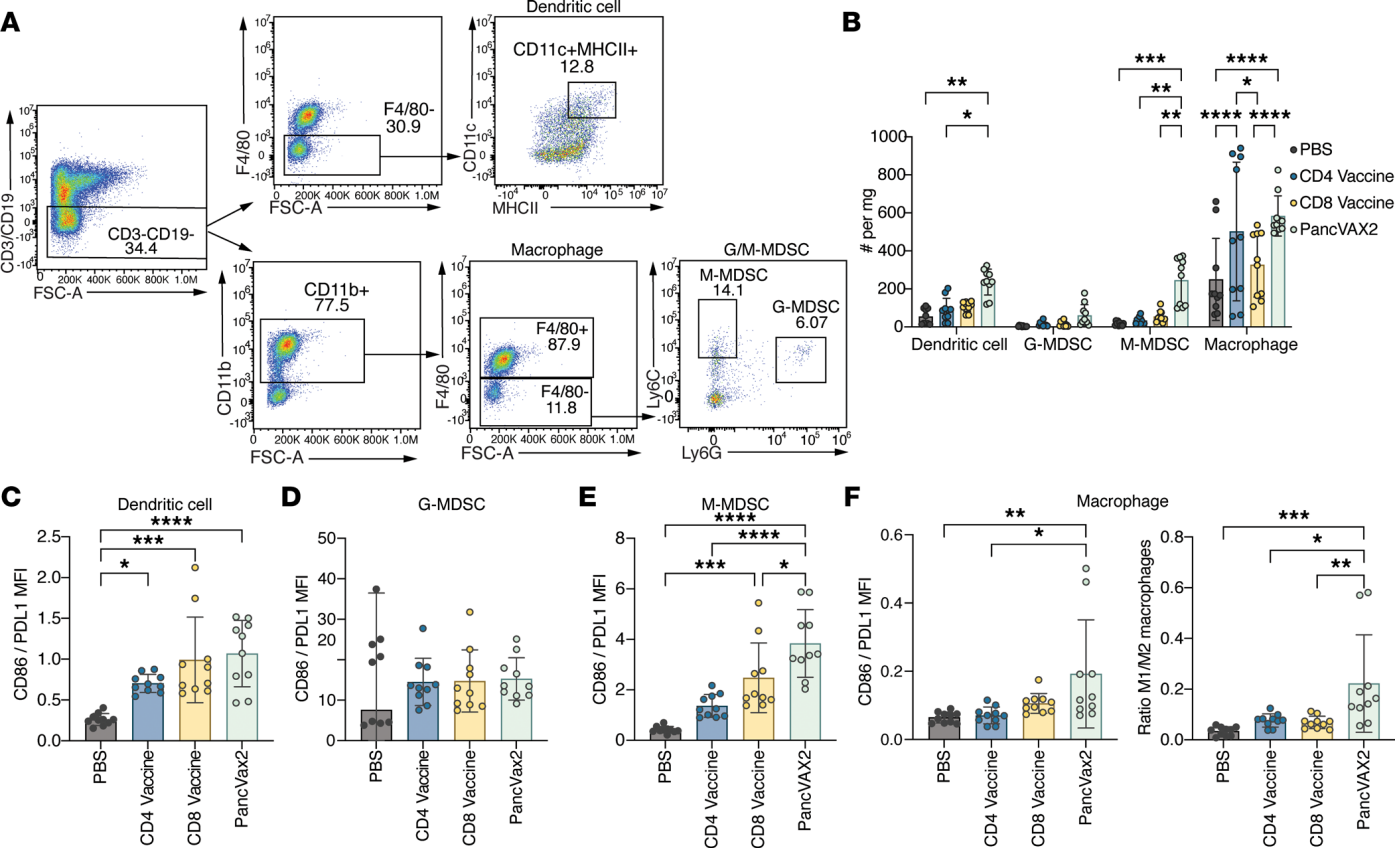
PancVAX2 induces proinflammatory TME changes in the myeloid population. C57BL/6 mice were implanted on the right flank, s.c., with 3 × 10^6^ Panc02 cells on day 0. Mice were vaccinated s.c. at the base of the tail on day 14 and 21 with PBS, CD4 vaccine, CD8 vaccine, or PancVAX2 (*n* = 5). On day 28, tumors were harvested, dissociated into a single-cell suspension, and stained for flow cytometry analysis. (**A**) Representative gating strategy for intratumoral myeloid cell populations. From the live CD45^+^ population, CD3^+^ or CD19^+^ cells were excluded. To identify DCs, MHCII^+^CD11c^+^ cells were gated on from the F4/80^–^ population of the CD3^–^CD19^–^ gate. Macrophages were gated as CD11b^+^F4/80^+^ cells from the CD3^–^CD19^–^ gate. Of the F4/80^–^ population Ly6C^hi^ M-MDSCs and Ly6G^+^Ly6C^lo^ G-MDSCs were identified. (**B**) Quantification of each myeloid subpopulation per mg tumor for each vaccine group. (**C**–**F**) To evaluate the proinflammatory or tumor suppressive nature of each myeloid subpopulation, the ratio of CD86/PD-L1 expression by MFI was determined on DCs (**C**), G-MDSCs (**D**), M-MDSCs (**E**), and macrophages (left) or the M1/M2 ratio of tumor infiltrating macrophages (right) (**F**) was determined by evaluating the proportion of CD86^+^ to CD206^+^ macrophages. Symbols represent technical duplicate of individual mice (*n* = 5). Data are shown as mean ± SD. Significance was calculated by 2-way ANOVA followed by Sidak’s multiple-comparison test. *P ≤ 0.05, **P ≤ 0.01, ***P ≤ 0.001, ****P ≤ 0.0001.

The reduction in immunosuppressive Tregs in the TME associated with Th1-like CD4^+^ T cell activation led us to determine whether PancVAX2 also alters myeloid cell populations. We quantified infiltrating myeloid cells within tumors after mice were vaccinated with PBS, CD8 vaccine, CD4 vaccine, or PancVAX2 (Figure 6, A and B). There was a significant increase in the number of DCs and monocytic myeloid derived suppressor cells (M-MDSCs) in mice treated with PancVAX2 compared with mice treated with the CD4 or CD8 vaccines alone (Figure 6B). Interestingly, macrophage numbers were significantly greater with both PancVAX2 and the CD4 vaccine compared with the CD8 vaccine or PBS, suggesting a CD4^+^ response–specific effect on macrophage recruitment into the TME.

We then assessed the expression of CD86 relative to PD-L1 on each cell type to determine their proinflammatory versus immunosuppressive function, respectively. All vaccine platforms increased the proinflammatory function of the DC compartment (Figure 6C), while no effect was observed in the granulocytic-myeloid derived suppressor cell (G-MDSC) population (Figure 6D). However, monocytic-myeloid derived suppressor cells (M-MDSCs) from mice vaccinated with PancVAX2 had the greatest increase in the ratio of CD86/PD-L1 expression (Figure 6E). Similarly, and most notably, vaccination with PancVAX2 led to an increased ratio in proinflammatory M1/immunosuppressive M2 macrophages (Figure 6F), demonstrating that the inclusion of both CD4 and CD8 neoantigens is important for effective repolarization of monocytes and macrophages, leading to broad scale changes in the antitumor phenotype of the TME.

## Discussion

Pooled peptide vaccines targeting multiple neoantigens in a patient’s tumor have been preclinically and clinically utilized to help overcome neoantigen loss and increase the potential of activating antitumor T cell responses, since only a fraction of predicted neoantigens are found to be immunogenic (3–5, 18, 19). Our study establishes that the inclusion of CD4^+^ T cell–activating neoantigens in a personalized vaccine is salient for optimal antitumor efficacy. It also confirms that, while our previous neoantigen vaccine activates primarily CD8^+^ T cell responses, it requires the addition of immune checkpoint modulators for the effective control of tumor growth. In contrast, PancVAX2, which contains both CD4 and CD8 neoepitopes, provides a significant reduction in tumor growth and a long-term survival benefit without concomitant checkpoint modulation. This study provides rationale for prioritizing both CD4 and CD8 activating neoantigens in a single vaccine but also underpins the biological importance of CD4^+^ T cell help in the context of personalized vaccines that are being considered for testing in patients.

While CD4^+^ T cells have multiple known roles in antitumor immunity, they have not yet been prioritized for targeting tumor neoantigens in most vaccination strategies. Our study provides evidence for several notable mechanisms through which the inclusion of CD4 epitopes enhance antitumor immunity. We observed that concomitant vaccination of CD4^+^ and CD8^+^ epitopes not only improved the activation of neoantigen-specific CD8^+^ T cell responses but also enhanced the recruitment of cytotoxic CD8^+^ T cell effectors to the TME. Interestingly, there was a significant increase in tumor infiltration of certain neoantigen-specific CD8^+^ T cells, including those specific to peptides 44, 66, 77, 175, and 219 but not for peptide 237. Tumor-specific CD4^+^ T cells have been shown to promote CXCL9/10 expression through IFN-γ–dependent mechanisms that increase CD8^+^ T cell recruitment, particularly of low-affinity T cell receptor (TCR) CD8^+^ T cell clones (20, 21). Future efforts aim to delineate how T cell TCR affinity for each of the CD8 neoantigens may influence trafficking and retention with the TME. We also found that neoantigen-specific CD8^+^ T cells within the TME had lower PD-1 expression after cognate antigen recognition with PancVAX2 vaccination, suggesting less neoantigen-specific CD8^+^ T cell exhaustion in the TME. This is consistent with a known role of CD4^+^ T cell engagement with DCs resulting in the upregulation of CD70 on DCs; CD70 acts as a costimulatory molecule for CD27 on CD8^+^ T cells to promote a T effector transcriptional signature with downregulated exhaustion markers (22).

PancVAX2-induced CD4^+^ T cells did not express the cytotoxic effector molecule GzmB, suggesting that the CD4^+^ T cells were not directly cytolytic, as has been reported preclinically and clinically in a subset of tumor-reactive T cells (23–25). However, IFN-γ–producing CD4^+^ T cells can both indirectly and directly kill tumor cells (26–28). MHCII was upregulated in Panc02 cells after in vitro IFN-γ treatment; thus, this mechanism is not entirely excluded. IFN-γ–mediated cytotoxicity may occur through either direct CD4^+^ tumor cell recognition or by APC presentation in the TME. CD4^+^ T cell engagement with antigen-presenting macrophages has also been shown to promote indirect tumor cytotoxicity of reactive oxygen species (29). Finally, while our study demonstrates that a CD4^+^ Th1 response is important for improving the therapeutic efficacy of neoantigen vaccines, other Th subtypes may provide additional therapeutic benefit. Toward this, the choice of immune adjuvant may play a role in differentiating specific CD4^+^ Th subtypes. We utilized ADU-S100, a second-generation synthetic cyclic dinucleotide that potently activates STING to signal through IRF3, resulting in high-magnitude CD8^+^ T cell and CD4^+^ Th1 responses (30, 31). Use of or codelivery of adjuvants that promote alternative CD4^+^ T cell fates such as Tfh and germinal center formation, with GLA-SE or A-910823, could enhance formation of tertiary lymphoid structures, which are highly associated with improved therapeutic benefit of cancer vaccines (32–34). Understanding the optimal adjuvants that induce high-quality and durable antitumor responses remains to be further explored.

We also found that the activation of CD4^+^ T cell responses by PancVAX2 was required for reprogramming of the immunosuppressive TME. Infiltration of neoantigen-specific Th1 CD4^+^ T cells within the tumor was associated with a significant reduction in FOXP3^+^ Tregs. Additionally, although there was significantly greater infiltration of M-MDSCs into the TME after PancVAX2 vaccination, these cells had a significantly less immunosuppressive protein expression signature with high CD86 to PD-L1 expression. This is consistent with previous reports of type I IFN– and TNF-α–mediated inhibition of M-MDSC function by activated T cells (35). Most notably, there was a greater M1 to M2 macrophage ratio, suggesting repolarization to an antitumor macrophage phenotype. Consistent with these data, Th1 CD4^+^ T cells have been reported to instruct macrophage populations to transition to an M1-like phenotype through IFN-γ signaling (36). Future single-cell RNA-Seq studies should delineate differentially regulated pathways in the myeloid and lymphoid populations in the context of CD4^+^ T cell activation.

Many personalized neoantigen vaccines tested clinically have utilized SLPs of neoantigen targets and demonstrated a predominant CD4^+^-specific T cell response (3–5). However, the relative therapeutic importance of activation of these CD4^+^ T cell responses has been unclear. Importantly, these Phase I studies were not powered to assess clinical responses and were conducted in the adjuvant setting where there is no visible disease. Although little can be gleaned from the clinical responses, these studies demonstrate the induction of poly-functional neoantigen-specific T cell responses that were still detectable 4 years after vaccination in patients with advanced melanoma (3). A distinct difference between the clinical application of personalized vaccines and our model system was our ability to functionally screen neoantigens for CD8^+^ and CD4^+^ T cell responses by IFN-γ ELISpot specific to the Panc02 tumor line. This allowed us to prioritize the most immunogenic CD8 and CD4 activating neoantigens, which did not correlate with predicted binding affinity determined by NetMHC. Interestingly, we found that CD8-specific neoantigen immunogenicity correlated with predicted structural features of neoantigen display (16). Since most clinical trials utilize NetMHC or similar algorithms to prioritize neoantigens, there may be limitations to the identification of the strongest CD8 and CD4, and this may have greater specificity to their response. To this point, newer algorithms of neoantigen prediction that integrate biophysical features of neoantigen quality and TCR engagement as a predictive feature of immunogenicity have improved identification of strong neoantigen targets (37, 38). Recent application of these improved algorithms to generate a personalized neoantigen vaccine for patients with resected pancreatic cancer demonstrated strong activation of neoantigen-specific CD8^+^ T cells (19). Future studies that improve these analyses in the context of MHCII-specific CD4^+^ T cell neoantigens may further enhance these overall responses.

Predicting immunogenic MHCII-specific neoantigens that should be included in personalized cancer vaccines remains a challenge. We generated a ranked list of potential MHCII binding epitopes using IEDB and employed a liberal cut-off for epitopes ranked in the top 50% of predicted binding affinity. These were then screened for in vivo immunogenicity to yield 6 CD4^+^ T cell–activating neoantigens for PancVAX2. Interestingly, NetMHCpan4.0 only predicted weak binders in the context of H2-IA^b^ for 3 of the 6 immunogenic CD4^+^ T cell neoantigens, underscoring the need for improved methods of MHCII neoantigen identification. Indeed, machine learning methods from mass spectrometry data of MHCII alleles have improved the identification of immunogenic epitopes (39). Building on this, methods that integrate mass spectrometry–based binding data, antigen expression levels, and proteolytic cleavage predictions, such as MARIA, have recently demonstrated the enhanced ability to better predict MHCII immunogenic targets (40). Subcellular protein localization has also been identified as an important contributor to MHCI or MHCII epitope presentation and improves predictions for immunogenic neoantigens (41). Importantly, neoantigen identification methods that incorporate measurements of distance from self, thereby ensuring targets that do not activate tolerogenic or Treg responses, are essential for effective therapeutic outcomes.

In this study, we determined that inclusion of MHCII-specific neoantigens in personalized cancer vaccines is critical for optimal priming and recruitment of cytotoxic CD8^+^ T cells to the tumor. Additionally, these CD4^+^ T cell responses play an important role in remodeling the TME to a proimmune environment. This work sets the stage for future development of optimized CD4^+^-specific and CD8^+^-specific T cell responses in a personalized vaccine approach that will be essential for successful therapeutic outcomes in hard-to-treat cancer types such as pancreatic cancer.

## Methods

### Mice and study approval.

Five- to 6-week-old male C57BL/6 mice were purchased from The Jackson Laboratory and were allowed to acclimate for 1 week prior to the experiments.

### Cell cultures.

The murine pancreatic cancer cell line, Panc02, was maintained in DMEM supplemented with 10% FBS (Gemini Bio Products), 1% L-Glutamine (Thermo Fisher Scientific, catalog 25030-081), 0.5% penicillin/streptomycin (Thermo Fisher Scientific, catalog 15140-122) at 37°C in 10% CO_2_. T2-H2-K^b^, T2-H2-D^b^, and T2-IA^b^ cells were maintained in RPMI supplemented with 10 % FBS, 1% L-Glutamine (Thermo Fisher Scientific), 1% penicillin/streptomycin (Invitrogen), 1% MEM Non-Essential Amino Acids (Thermo Fisher Scientific, catalog 11140-050), and 1% sodium pyruvate (Thermo Fisher Scientific, catalog 11360-070). T2-H2-K^b^ and T2-H2-D^b^ cell lines and the T2-IA^b^ cell line were subject to selection in 1 mg/mL and 0.25 mg/mL Geneticin (Thermo Fisher Scientific, catalog 10131-027), respectively. All cell lines were confirmed to remain free of *Mycoplasma* through regular testing at the Johns Hopkins University Genetic Resources Core Facility.

Primary murine splenocytes or TILs were maintained in RPMI supplemented with 10% FBS, 0.5% L-glutamine, 1% penicillin/streptomycin, and 1× BME (Thermo Fisher Scientific, catalog 21985-023) at 37°C in 5%CO_2_.

Primary murine BMDCs were prepared by isolating bone marrow from non–tumor-bearing C57BL/6 mice per the Jove protocol (42). Isolated bone marrow was treated with ACK lysis buffer, washed with complete lymphocyte media, and then resuspended at 0.5 × 10^6^ cells/mL in media supplemented with 20 ng/mL murine GM-CSF (PeproTech, catalog 315-03). Cells were seeded at 1 × 10^6^ cells/well in 24-well plates. Media were replenished with fresh GM-CSF–containing media on day 3. BMDCs were matured with 100 ng/mL LPS on days 4 and 5, collected on the next day, and analyzed by flow cytometry to confirm expression of CD11c, MHCII, and CD86 expression.

### MHCII epitope prediction.

We previously screened and generated SLPs for 245 neoantigens identified in the Panc02 line that were predicted to bind 2 MHCI alleles expressed in Panc02 cells, namely H2-K^b^ or H2-D^b^, with a binding affinity of < 1,000 nM (14). To build on this, we used IEDB to rank the binding affinity of these 245 neoantigens to I-A^b^, the MHCII allele expressed by Panc02. Neoepitopes that displayed a binding affinity greater than the 50th percentile for I-A^b^ were tested for in vivo immunogenicity.

### Peptide vaccines.

Peptides were synthesized by Peptide 2.0 at 95% purity. Lyophilized peptides were stored at –20°C with CaSO_4_ desiccant (Drierite). Peptides were dissolved in DMSO at 50 mg/mL, aliquoted, and stored at –80°C. Peptide vaccines were prepared by diluting AddaVax at a 1:1 vol/vol ratio with peptides and 2’3’-c-di-AMP(PS)_2_ (Rp,Rp), an analog of c-di-AMP STING agonist (5 μg/dose, InvivoGen, catalog vac-nacda2r) in PBS, vortexed 3 times, and incubated for 10 minutes. Peptides were added to the Addavax/STING/PBS mix at a final concentration of 50 μg/peptide and vortexed 3 times. Peptide vaccine composition was as follows: CD4 vaccine (peptides 43, 53, 133, 197, 230, and 239), CD8 vaccine (peptides 20, 23, 44, 66, 77, 84, 94, 175, 219, and 237), and PancVAX2 (peptides 20, 23, 43, 44, 53, 66, 77, 84, 94, 133, 175, 197, 219, 230, 237, and 239). Sequences shown in Supplemental Tables 1 and 2.

### In vivo immunogenicity studies.

Six- to 7-week-old non–tumor-bearing C57BL/6 mice were vaccinated with CD4 vaccine, CD8 vaccine, or PancVAX2 or PBS twice, 7 days apart. Mice were injected with 100 μL of vaccine s.c. at the base of the tail. Seven days following the second vaccination, spleens were harvested for downstream analysis of T cells.

### Lymphocyte isolation from spleens.

Freshly harvested spleens were processed into a single-cell suspension by passing the spleen through a 40 μm filter (CELLTREAT, catalog 229481) utilizing a pestle (CELLTREAT, catalog 229480). Single-cell suspensions were centrifuged at 428 g for 5 minutes, supernatant aspirated, and then resuspended in 1 mL ACK lysis buffer for 2 minutes at room temperature. In total, 10 mL splenocyte medium was added to quench ACK lysis buffer. CD8^+^ or CD4^+^ T cells were negatively isolated using EasySep Mouse CD8^+^ or CD4^+^ T cell Isolation Kits (STEMCELL Technologies; CD8 Kit, catalog 19853; CD4 Kit catalog 19852) per manufacturer’s protocol. Purified CD4^+^ and CD8^+^ T cell populations were verified by flow cytometry for CD4 and CD8 expression.

### In vivo tumor studies.

Five- to 6-week-old C57BL/6 mice were implanted s.c. with 3 × 10^6^ Panc02 cells with a 30 gauge insulin needle. For tumor immune cell analysis, tumors were allowed to establish for 14–28 days. Mice were then vaccinated with CD4 vaccine, CD8 vaccine, or PancVAX2 or PBS s.c. twice, 7 days apart. Seven days after the second vaccine dose, mice were euthanized and tumors were isolated for downstream analysis. For survival studies, tumor-bearing mice were vaccinated s.c. on days 3 and 10. Tumor growth was measured by calipers, and mice bearing tumors that reached 10 mm by any measurement were euthanized.

For PancVax with combination ICI tumor growth and survival studies, 6- to 7-week-old C57BL/6 mice were implanted s.c. with 3 × 10^6^ Panc02 cells with a 30 gauge insulin needle. Mice were given 1 of the following treatment regimens: isotype antibodies, anti–CTLA-4 (InVivoMAb BioXCell, catalog BE0131) + anti–PD-1 (InVivoMAb BioXCell, catalog BE0146) antibodies, PancVAX + isotype antibodies, or PancVAX + anti–CTLA-4 + anti–PD-1. Tumor-bearing mice were vaccinated s.c. on day 12 and 19 with PancVAX or PBS. PancVAX includes peptides 20, 23, 44, 66, 77, 84, 94, 175, 218, 219, 230, and 237. Vaccines for the tumor growth and survival with PancVAX and ICI were prepared as detailed above. Vaccines for the single-cell RNA-seq experiment were prepared as detailed above, with ADU V16 as the sting agonist at a dose of 5 μg/mouse. Mice were dosed with InVivoMAb anti-mouse (100 μg, BioXCell, catalog BE0146) PD-1 or InVivoMAb rat IgG2a isotype control (100 μg, BioXCell, catalog BE0090) starting at day 12. Mice were dosed with InVivoMAb anti–mouse CTLA-4 antibodies (100 μg, BioXCell, catalog BE0131) or isotype InVivoMAb polyclonal Syrian Hamster IgG (100 μg, BioXCell, catalog BE0087) were dosed on day 15 and day 22. Tumor growth was measured by calipers, and mice bearing tumors that had ulcerated or reached 10 mm by any measurement were euthanized.

### TIL isolation.

Tumors were processed to a single-cell suspension using a mouse tumor dissociation kit (Miltenyi Biotec, catalog 130-096-730) and gentleMACS dissociator. Briefly, tumors were collected and weighed before being chopped into small fragments and then transferred to a Miltenyi gentleMACS C tube (Miltenyi Biotec, catalog 130-093-237). Media containing diluted tumor dissociated kit enzymes were added to the tube. Tumors were digested in the gentleMACS dissociator with the “soft tumor” setting for 40 minutes. Once dissociated, tumors were filtered first using a 100 μm filter (CELLTREAT, catalog 229485), and then using a 40 μm filter (CELLTREAT, catalog 229481). Single-cell suspensions were centrifuged at 428 g for 5 minutes; supernatant was aspirated and then resuspended in 1 mL ACK lysis buffer for 1 minute at room temperature. In total, 10 mL of the splenocyte medium was added to quench ACK lysis buffer. Cells were centrifuged at 428 g for 5 minutes; supernatant was aspirated and resuspended in splenocyte media. For whole TIL analysis, 15 or 30 mg of tumor was plated.

CD8^+^ and CD4^+^ T cells were successively isolated from processed tumors. First, mouse CD8^+^ T cells were positively isolated using CD8^+^ TIL microbeads (Miltenyi Biotec, catalog 130-116-478) per the manufacturer. Flow through was collected, and CD4^+^ T cells were positively isolated using CD4^+^ TIL microbeads (Miltenyi Biotec, catalog 130-116-475) per manufacturer. Purified CD4^+^ and CD8^+^ T cell populations were verified by flow cytometry for CD4 and CD8 expression.

### Single-cell RNA-Seq.

Mice were implanted with tumors and treated following the same PancVax treatment regimen previously described above (14). Mice were sacrificed at 38 days after Panc02 implantation. Tumors were dissociated as described above. Samples were sequenced in 2 batches, one composed of single-cell suspensions from dissociated tumors pooled within the same treatment groups and one immune-enriched single-cell suspensions from pooled dissociated tumor cells using Percoll (MilliporeSigma) density separation. Library preparation was conducted using Chromium Single Cell 5′ Library and Gel Bead Kit followed by NextSeq 500 sequencing (Illumina). Sequence alignment and counting was conducted with CellRanger (v 4.0.0). Data analysis was carried out in R (v. 4.2.0) using Seurat (v. 4.1.1). Genes were filtered to include only genes expressed in at least 3 cells. Cells were filtered to include only cells with between 200 and 3,000 genes detected, between 1,000 and 10,000 unique molecular identifiers (UMI), less than 10% of UMIs from mitochondrial genes, and no expression of hemoglobin genes. Cell cycle G2M and S phase scores were calculated using the Seurat module score function and mouse orthologs of the human cell cycle-associated genes from Kowalczyk et al. (43) Expression counts were normalized by log transformation with a scaling factor of 10,000 and scaled with regressing out of G2M and S phase scores. Batch correction of the low-dimensional embeddings of cells with regression of the 2 sequencing batches was carried out using Harmony (v. 0.1.0). (44) Neighborhoods of cells were detected using the Seurat FindNeighbors function based on the first 25 Harmony dimensions. Uniform manifold approximation projection (UMAP) embeddings were calculated based on the first 25 dimensions from Harmony with embedding parameters a = 0.9922, b = 1.112. Clusters of transcriptionally similar cells were detected with Louvain clustering at resolution 0.6. Clusters of cells were annotated based on expression of canonical marker genes. Louvain clustering at resolution 1.0 was used to distinguish NK cells from naive CD8^+^ T cells. Differential gene expression between treatment groups was assessed using the MAST test wrapper of the Seurat FindMarkers function controlling for batch. Tests considering all CD8^+^ T cells used cells annotated as cycling_CD8_T, cytotoxic_CD8_T, exhausted_CD8_T, or naïve_CD8_T. Results of MAST differential gene expression tests were plotted using EnhancedVolcano (v. 1.14.0). T cell phenotype proportions were calculated for each treatment group as the number of cells annotated as the T cell phenotype divided by the total number of T cells within the treatment group. Stacked bar plots were rendered using ggplot2 (v. 3.4.1).

### ELISpot.

Capture antibody, anti–mouse IFN-γ (clone AN18, Mabtech, catalog 3321-3-1000), was diluted (1:100 in PBS) and added (100 μL) to each well of the filter plate (MilliporeSigma, catalog MSHAS4B10). Plates were covered with parafilm and incubated overnight at 4°C. The following morning, plates were washed 3 times with PBS and blocked with complete lymphocyte media for at least 2 hours at 37°C. T2-H2-K^b^, T2-H2-D^b^, T2-I-A^b^, or BMDCs were used as APCs, which were collected, washed with PBS, counted, and resuspended in lymphocyte media at 1 × 10^6^ cells/mL. Individual peptides were added at a final concentration of 2.5 μg/mL and shaken for 2 hours at room temperature. CD8^+^ or CD4^+^ T cells, isolated as described above, were resuspended in lymphocyte media at 1 × 10^6^ cells/mL. In total 1 × 10^5^ peptide-pulsed APCs and 1 × 10^5^ T cells were added in a total volume of 200 μL to the coated and blocked ELISpot plate and incubated overnight at 37°C. The following day, plates were washed 6 times with PBST and incubated with 100 μL secondary anti–mouse IFN-γ biotin (clone R4-6A2-biotin, Mabtech, catalog 3321-6-1000) diluted 1:1,000 in PBS for 2 hours at room temperature. Thirty minutes before incubation with the secondary antibody was complete, Vectastain ABC-HRP Kit (Vector Laboratories, catalog PK-4000) was prepared by diluting reagent A (1:100) and reagent B (1:100) in PBS, followed by vortexing. After a 30-minute incubation, plates were washed 6 times with PBST and 100 μL of prepared Vectastain reagent was added to each well and incubated for 1 hour at room temperature. Plates were washed 3 times with PBST and PBS in succession. AEC substrate (Vector Laboratories, catalog SK-4200) was prepared by adding 144 μL reagent 1, 180 μL reagent 2, and 160 μL reagent 3 to 10 mL deionized (DI) water (for each plate), followed by vortexing between reagent addition. In total, 100 μL AEC substrate was added to each well and incubated for 15 minutes. Plates were washed 6 times with DI water and patted on paper towels to dry overnight at room temperature, with plastic bottoms removed. Spots were quantified in the immune-monitoring core at Johns Hopkins University.

### Antibodies.

The following antibodies were used for flow cytometry analysis: anti–CD45-AF700 (clone 30-F11, 1:500, BD Biosciences, catalog 560510); anti–CD11c-BV605 (clone N418, 1:250, BioLegend, catalog 117334); anti–IA/IE-BV650 (clone M5/114.15.2, 1:500, BioLegend, catalog 107641); anti–CD11b-FITC (clone M1/70, 1:250, BD Biosciences, catalog 553310); anti–F4/80-PECy7 (clone BM8, 1:500, Invitrogen, catalog 25-4801-82); anti–Ly6G-BV421 (clone IA8, 1:250, BD Biosciences, catalog 562737); anti–Ly6C-PECy5.5 (clone HK1.4, 1:250, BioLegend, catalog 128012); anti-CD86 (clone GL-1, 1/500, BioLegend, catalog 105012); anti–CD206-PE/Dazzle (clone C068C2, 1:100, BioLegend, catalog 141732); anti–PD-L1–PE (clone M1H5, 1:100, BD Biosciences, catalog 558091); anti–CD3-BV785 (clone 17A2, 1:250, BioLegend, catalog 100232); anti–CD19-BV785 (clone 6D5, 1:250, BioLegend, catalog 115543); anti–CD3-PE/Dazzle (clone 17A2, 1:500, BioLegend, catalog 100246); anti–CD8–PacBlue (clone 53-6.7, 1:500, BD Biosciences, catalog 558106); anti–CD4-BV605 (clone RM4-5, 1:500, BioLegend, catalog 100547); anti–NK1.1-BV650 (clone PK136, 1:100, BioLegend, catalog 108736); anti–CD44-APC (clone IM7, 1:250, BioLegend, catalog 103012); anti–PD-1–FITC (clone J43, 1:100, Invitrogen, catalog 11-9985-85); anti–CD137-PE (clone 17B5, 1:250, BioLegend, catalog 106105); anti–GzmB-PECy7 (clone NGZB, 1:100, Invitrogen, catalog 25-8898-82); anti–IFN-γ–PerCP/Cy5.5 (clone XMG1.2, 1:100, Invitrogen, catalog 45-7311-82); anti–IL-2–APC (clone JES6-5H4, 1:100, Invitrogen, catalog 17-7021-81); anti–TNF-α–AF700 (clone MP6-XT22, 1:100, BioLegend, catalog 506338); anti–CD4-PacBlue (clone RM4-5, 1:250, BioLegend, catalog 100531); anti–CD137-APC (clone 17B5, 1:250, BioLegend, catalog 106109); anti–IL-2–BV605 (clone JES6-5H4, 1:100, BioLegend, catalog 503829); anti–IL-10–AF488 (clone JES5-16E3, 1:100, BioLegend, catalog 505006), anti–IL-4–PE (clone 11B11, 1:100, BD Biosciences, catalog 554435), anti–GzmB-PE eFluor610 (clone NGZB, 1:100, Invitrogen, catalog 61-8898-82), anti–IL-2–BV605 (clone JES6-5H4, 1:100, BioLegend, catalog 503829), anti–IL-17A-BV650 (clone TC11-18H10.1, 1:100, BioLegend, catalog 506930), anti–FOXP3-AF488 (clone FJK-16s, Invitrogen, catalog 53-5773-82), anti–T-bet–PE/Cy7 (clone 4B10, BioLegend, catalog 644824), and anti–Gata-3–eFluor450 (clone TWAJ, Invitrogen, catalog 48-9966-41).

### Flow cytometry.

Single-cell suspensions were plated in 96-well U-bottom plates. For samples with cytokine detection, cells were treated with 1× Protein Transport Inhibitor Cocktail (eBioscience, catalog 00-4980-03) for at least 5 hours prior to collection and staining. Cells were washed twice with PBS and stained for viability in 100 μL of Zombie NIR fixable live/dead stain (1:1500, BioLegend, catalog 423106) and Mouse BD Fc Block (1:50, BD Biosciences, catalog 553141) in PBS for 15 minutes at room temperature. Cells were washed twice with FACS buffer (1× HBSS, 2% FBS, 0.1% Sodium Azide) and then stained with 100 μL extracellular antibody mix diluted in FACS buffer for 20 minutes at 4°C. The cells were then washed twice with FACS buffer. For intracellular cytokine detection, BD Cytofix/Cytoperm Fixation/Permeabilization Kit was used (BD Biosciences, catalog 554714). For transcription factor detection, eBioscience FOXP3/Transcription Factor Staining Buffer Set was used (Invitrogen, catalog 00-5523-00). Briefly, cells were permeabilized in 100 μL fix/perm for 20 minutes at 4°C, washed twice in 1× perm/wash buffer, and then stained with 100 μL intracellular antibody mix diluted in 1× perm/wash buffer for 30 minutes at 4°C. Cells were then washed twice with 1× perm/wash buffer and were immediately run on a BD Cytoflex. All flow cytometry data were analyzed using FlowJo version 10.8.1.

### Statistics.

Data were graphed and analyzed by GraphPad Prism (version 9.5.1). Multiple comparisons were analyzed by 1-way or 2-way ANOVA, followed by Hidak-Holms or Dunnett’s test when significance was found. Survival studies were analyzed by a log-rank test. Data are represented as mean ± SD. Tumor growth plots are represented as mean ± SEM. *P* ≤ 0.05 is considered significant.

### Study approval.

All animal studies were approved by the IACUC at Johns Hopkins University.

### Data availability.

All investigators are committed to timely distribution of data obtained in this research work. The data discussed in this publication have been deposited in NCBI’s Gene Expression Omnibus (45) and are accessible through GEO Series accession no. GSE244992. All programming code used in analysis and computational tool development will be shared as open-source software via GitHub and Bioconductor at time of publication at https://github.com/FertigLab/PacVAX2_pancvaxICI_scRNAseq, commit ID 69053b3. Values for all data points in graphs are reported in the Supporting Data Values file.

## Author contributions

Study design was performed by ALH, TDA, EDM, MY, NSA, EJF, NZ, EMJ, and NZ. Experimental design was developed by ALH, TDA, GL, LA, EDM, FC, and NZ. Data acquisition was performed by ALH, LA, TDA, GL, MRL, BB, SR, LTK, EDM, SY, and RW. Data analysis and review was performed by ALH, TDA, GL, JTM, EDM, and EJF. Manuscript preparation was done by ALH, GL, JM, EJF, EMJ, and NZ. Man-uscript review was done by ALH, TDA, GL, JTM, LA, EDM, FC, RW, MRL, JM, BB, SR, JL, MY, NSA, SY, LTK, EJF, EMJ, and NZ.

## Supplementary Material

Supplemental data

Supporting data values

## Version 1 12/08/2023

Electronic publication

## Version 2 12/08/2023

Corrected figure

## References

[1] Xie N (2023). Neoantigens: promising targets for cancer therapy. Signal Transduct Target Ther.

[2] Yarchoan M (2017). Targeting neoantigens to augment antitumour immunity. Nat Rev Cancer.

[3] Hu Z (2021). Personal neoantigen vaccines induce persistent memory T cell responses and epitope spreading in patients with melanoma. Nat Med.

[4] Keskin DB (2019). Neoantigen vaccine generates intratumoral T cell responses in phase Ib glioblastoma trial. Nature.

[5] Ott PA (2017). An immunogenic personal neoantigen vaccine for patients with melanoma. Nature.

[6] Ott PA (2020). A phase Ib trial of personalized neoantigen therapy plus anti-PD-1 in patients with advanced melanoma, non-small cell lung cancer, or bladder cancer. Cell.

[7] Sahin U (2017). Personalized RNA mutanome vaccines mobilize poly-specific therapeutic immunity against cancer. Nature.

[8] Sahin U (2020). An RNA vaccine drives immunity in checkpoint-inhibitor-treated melanoma. Nature.

[9] Castle JC (2012). Exploiting the mutanome for tumor vaccination. Cancer Res.

[10] Hilf N (2019). Actively personalized vaccination trial for newly diagnosed glioblastoma. Nature.

[11] Jurtz V (2017). NetMHCpan-4.0: improved peptide-MHC class I interaction predictions integrating eluted ligand and peptide binding affinity data. J Immunol.

[12] Yu M, Levine SJ (2011). Toll-like receptor, RIG-I-like receptors and the NLRP3 inflammasome: key modulators of innate immune responses to double-stranded RNA viruses. Cytokine Growth Factor Rev.

[13] Ammi R (2015). Poly(I:C) as cancer vaccine adjuvant: knocking on the door of medical breakthroughs. Pharmacol Ther.

[14] Kinkead HL (2018). Combining STING-based neoantigen-targeted vaccine with checkpoint modulators enhances antitumor immunity in murine pancreatic cancer. JCI Insight.

[15] Fleri W (2017). The Immune epitope database and analysis resource in epitope discovery and synthetic vaccine design. Front Immunol.

[16] Zaidi N (2020). Role of in silico structural modeling in predicting immunogenic neoepitopes for cancer vaccine development. JCI Insight.

[17] Trinchieri G (2007). Interleukin-10 production by effector T cells: Th1 cells show self control. J Exp Med.

[18] Anagnostou V (2017). Evolution of neoantigen landscape during immune checkpoint blockade in non-small cell lung cancer. Cancer Discov.

[19] Rojas LA (2023). Personalized RNA neoantigen vaccines stimulate T cells in pancreatic cancer. Nature.

[20] Bos R, Sherman LA (2010). CD4+ T-cell help in the tumor milieu is required for recruitment and cytolytic function of CD8+ T lymphocytes. Cancer Res.

[21] Wong SB (2008). Tumor-specific CD4+ T cells render the tumor environment permissive for infiltration by low-avidity CD8+ T cells. J Immunol.

[22] Ahrends T (2017). CD4^+^ T cell help confers a cytotoxic T cell effector program including coinhibitory receptor downregulation and increased tissue invasiveness. Immunity.

[23] Cachot A (2021). Tumor-specific cytolytic CD4 T cells mediate immunity against human cancer. Sci Adv.

[24] Akhmetzyanova I (2013). Tumor-specific CD4+ T cells develop cytotoxic activity and eliminate virus-induced tumor cells in the absence of regulatory T cells. Cancer Immunol Immunother.

[25] Oh DY (2020). Intratumoral CD4^+^ T cells mediate anti-tumor cytotoxicity in human bladder cancer. Cell.

[26] Quezada SA (2010). Tumor-reactive CD4(+) T cells develop cytotoxic activity and eradicate large established melanoma after transfer into lymphopenic hosts. J Exp Med.

[27] Xie Y (2010). Naive tumor-specific CD4(+) T cells differentiated in vivo eradicate established melanoma. J Exp Med.

[28] Mumberg D (1999). CD4(+) T cells eliminate MHC class II-negative cancer cells in vivo by indirect effects of IFN-gamma. Proc Natl Acad Sci U S A.

[29] Kruse B (2023). CD4^+^ T cell-induced inflammatory cell death controls immune-evasive tumours. Nature.

[30] Corrales L (2015). Direct activation of STING in the tumor microenvironment leads to potent and systemic tumor regression and immunity. Cell Rep.

[31] Sivick KE (2018). Magnitude of therapeutic STING activation determines CD8^+^ T cell-mediated anti-tumor immunity. Cell Rep.

[32] Cui C (2021). Neoantigen-driven B cell and CD4 T follicular helper cell collaboration promotes anti-tumor CD8 T cell responses. Cell.

[33] Hill DL (2019). The adjuvant GLA-SE promotes human Tfh cell expansion and emergence of public TCRβ clonotypes. J Exp Med.

[34] Yoshioka Y (2023). A-910823, a squalene-based emulsion adjuvant, induces T follicular helper cells and humoral immune responses via α-tocopherol component. Front Immunol.

[35] Chen J (2021). Reprogramming immunosuppressive myeloid cells by activated T cells promotes the response to anti-PD-1 therapy in colorectal cancer. Signal Transduct Target Ther.

[36] Eisel D (2019). Cognate interaction with CD4^+^ T cells instructs tumor-associated macrophages to acquire M1-like phenotype. Front Immunol.

[37] Balachandran VP (2017). Identification of unique neoantigen qualities in long-term survivors of pancreatic cancer. Nature.

[38] Łuksza M (2022). Neoantigen quality predicts immunoediting in survivors of pancreatic cancer. Nature.

[39] Racle J (2019). Robust prediction of HLA class II epitopes by deep motif deconvolution of immunopeptidomes. Nat Biotechnol.

[40] Chen B (2019). Predicting HLA class II antigen presentation through integrated deep learning. Nat Biotechnol.

[41] Castro A (2022). Subcellular location of source proteins improves prediction of neoantigens for immunotherapy. EMBO J.

[42] Amend SR (2016). Murine hind limb long bone dissection and bone marrow isolation. J Vis Exp.

[43] Kowalczyk MS (2015). Single-cell RNA-seq reveals changes in cell cycle and differentiation programs upon aging of hematopoietic stem cells. Genome Res.

[44] Korsunsky I (2019). Fast, sensitive and accurate integration of single-cell data with Harmony. Nat Methods.

[45] Edgar R (2002). Gene expression omnibus: NCBI gene expression and hybridization array data repository. Nucleic Acids Res.

