# Supplementary material for: CD4 T cell–activating neoantigens enhance personalized cancer vaccine efficacy
Source: JCI Insight. 2023 Dec 8;8(23):e174027. doi: 10.1172/jci.insight.174027 (PMC10795827; doi:10.1172/jci.insight.174027)
Supplement: Supplemental data [file jciinsight-8-174027-s165.pdf]

Supplemental Figure 1

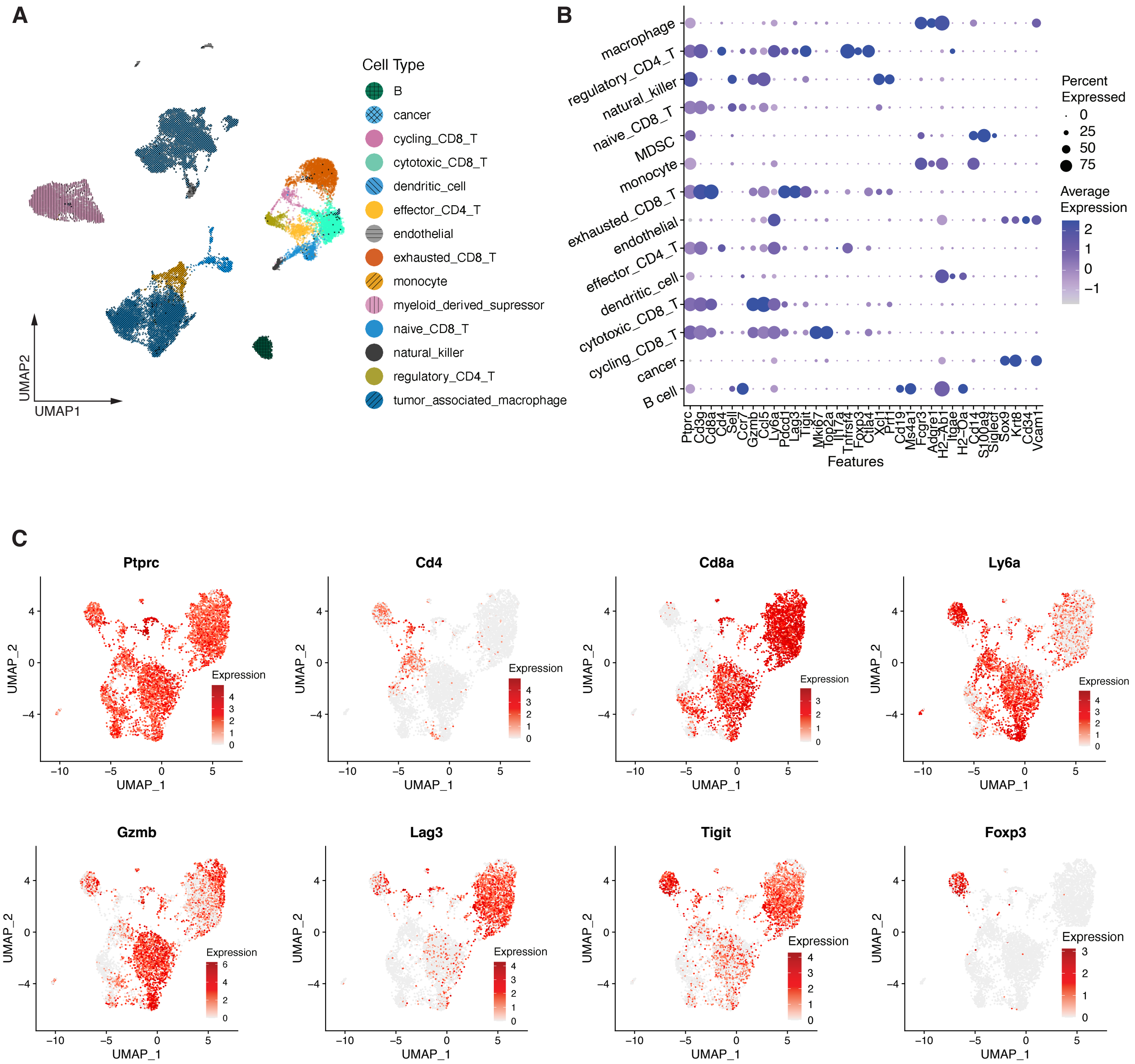

Supplemental Figure 2

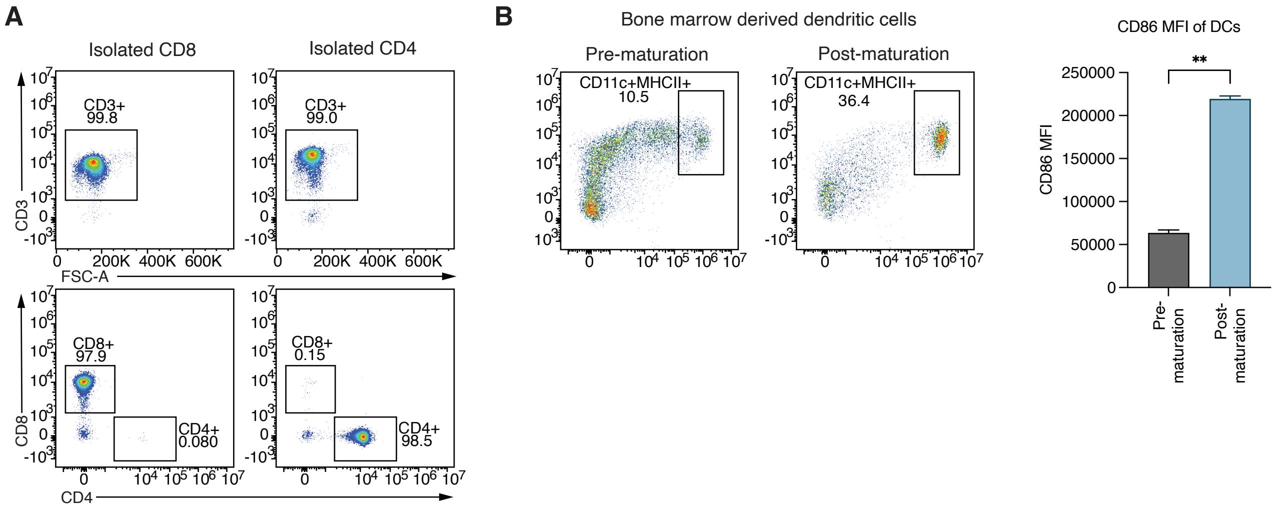

Supplemental Figure 3

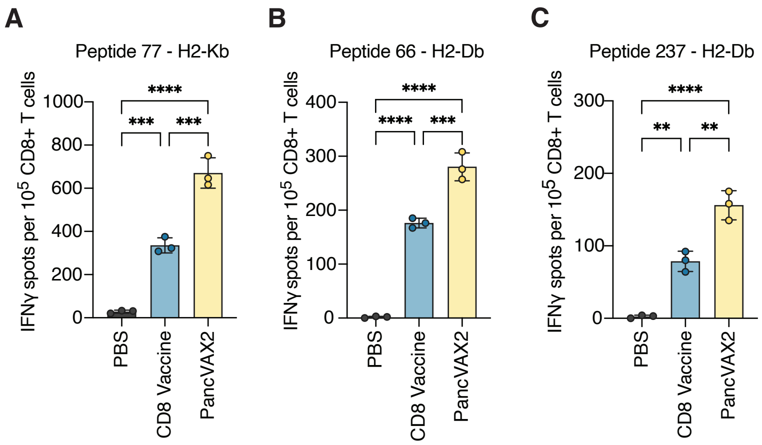

Supplemental Figure 4

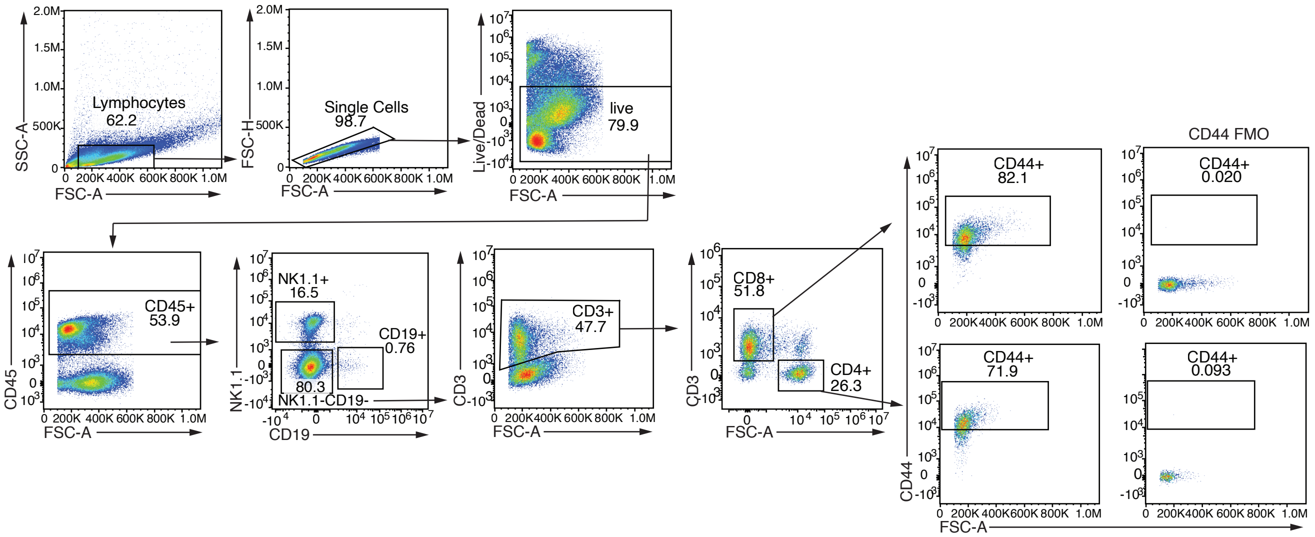

Supplemental Figure 5

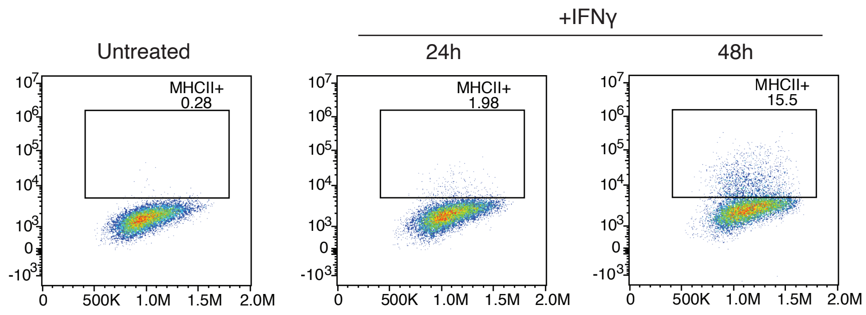

Supplemental Figure 6

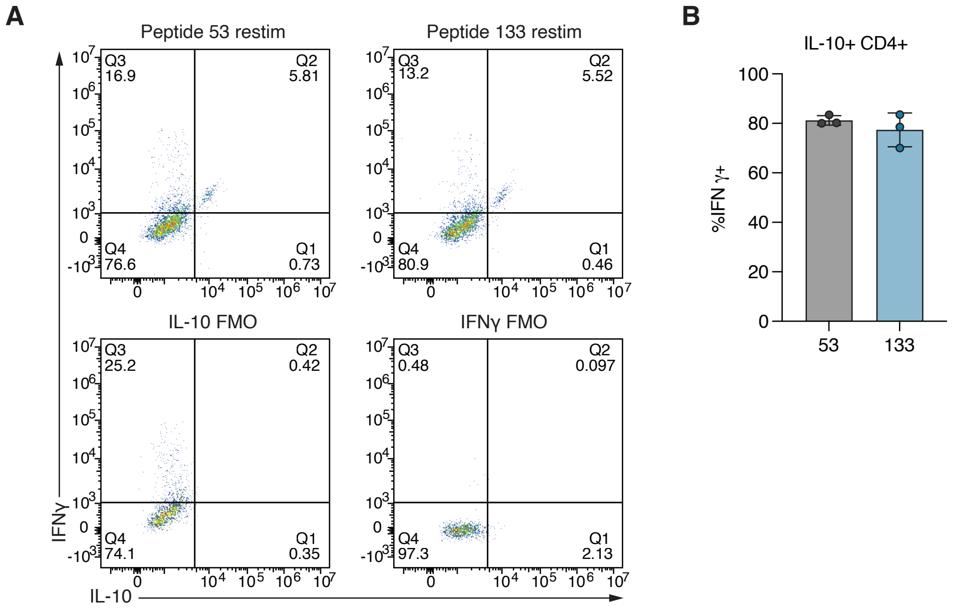

SUPPLEMENTAL TABLES

Supplemental Table 1: MHC Class I neoantigens used in CD8 Vaccine and PancVAX2

Peptide ID	Gene	Mutation	SLP sequence
20	Myo1g	K696N	LQGDVAFGHSNLFIRSPRTL
23	Ace	G473A	ALEKIAFLPFAYLVDQWRWG
44	Glb1l2	G36C	GSSAEESHLSCLNWSTLVPL
66	Map2k5	A11G	MLWLALGPFCGMENQVLVIR
77	Rasa3	T203S	TERIYSLFNLSMGKLEKMQE
84	Clcn7	D771G	ALGLRHLVVVGNHNQVVGLV
94	Notch2	A1969S	AELINCQADVSAVDDHGKSA
175	Bsg	A8P	MAAALLLPLAFTLLSGQ
219	Ppp2r3a	T197I	SHRNSLDTNLISMLFQNLSE
237	Ttn	E1901	IKIVRLTTGSAYQFRVCAEN

Supplemental Table 2: Immunogenic MHC Class II neoantigens identified in Panc02.
*Predicted weak binder for H2-IAb by NetMHCIIpan2.3

Peptide ID	Gene	Mutation	SLP sequence	Percentile Rank (IEDB)	Predicted binding affinity for highest rank 15-mer (nM)	Predicted binding affinity for highest rank 18-mer (nM)
43	Ankrd11	L2456V	YAEYVTYTGSVLLDGKPLSK	11.2	1289.1	820.2
53	Zfp955b	P23H	EDVAVNFSLGHWALLDSYQK	28.98	6287.0	3255.8
133	Kcnj12var2	G216A	DGKLCLMWRVANLRKSHIVE	16.06	1098.9*	509.3
197	Hmcn1	C2362	VHVSDTGRYVWVAVNVAGMT	1.88	348.3*	247.0
230	Tg	R2226	KVGTAWKQVYLFLGVPYAAP	9.36	2095.1	1345.1
239	Usp19	P829S	VQQRPQVPSISISKCAACQR	10.8	1116.0*	643.6
